# Development and validation of a novel N6-methyladenosine (m6A)-related multi- long non-coding RNA (lncRNA) prognostic signature in pancreatic adenocarcinoma

**DOI:** 10.1080/21655979.2021.1933868

**Published:** 2021-07-07

**Authors:** Qihang Yuan, Jie Ren, Lunxu Li, Shuang Li, Kailai Xiang, Dong Shang

**Affiliations:** a Department of General Surgery, First Affiliated Hospital of Dalian Medical University, Dalian, China; b Clinical Laboratory of Integrative Medicine, First Affiliated Hospital of Dalian Medical University, Dalian, China; c Department of Oncology, First Affiliated Hospital of Dalian Medical University, Dalian, China

**Keywords:** Pancreatic adenocarcinoma, N6-methyladenosine, long non-coding RNAs, prognostic signature, bioinformatics

## Abstract

Accumulating evidence has unveiled the pivotal roles of N6-methyladenosine (m6A) in pancreatic adenocarcinoma (PAAD). However, there are not many researches to predict the prognosis of PAAD using m6A-related long non-coding RNAs (lncRNAs). Raw data from The Cancer Genome Atlas (TCGA), International Cancer Genome Consortium (ICGC), and the Genotype-Tissue Expression project (GTEx) were utilized to comprehensively analyze the expression and prognostic performances of 145 m6A-related lncRNAs in PAAD and to develop and validate a novel m6A-related multi-lncRNA prognostic signature (m6A-LPS) for PAAD patients. In total, 57 differentially expressed m6A-related lncRNAs with prognostic values were identified. Based on LASSO-Cox regression analysis, m6A-LPS was constructed and verified by using five-lncRNA expression profiles for TCGA and ICGC cohorts. PAAD patients were then divided into high- and low-risKBIE_A_1933868k subgroups with different clinical outcomes according to the median risk score; this was further verified by time-dependent receiver operating characteristic curves. Risk scores were significantly associated with clinical parameters such as histological grade and cancer status among PAAD patients. A nomogram consisting of risk score, grade, and cancer status was generated to predict the survival probability of PAAD patients, as also demonstrated by calibration curves. Discrepancies in cellular processes, signaling pathways, and immune status between the high- and low-risk subgroups were investigated by functional and single-sample gene set enrichment analyses. In conclusion, the novel m6A-LPS for PAAD patients was developed and validated, which might provide new insight into clinical decision-making and precision medicine.

## GRAPHICAL ABSTRACT

**Figure:**
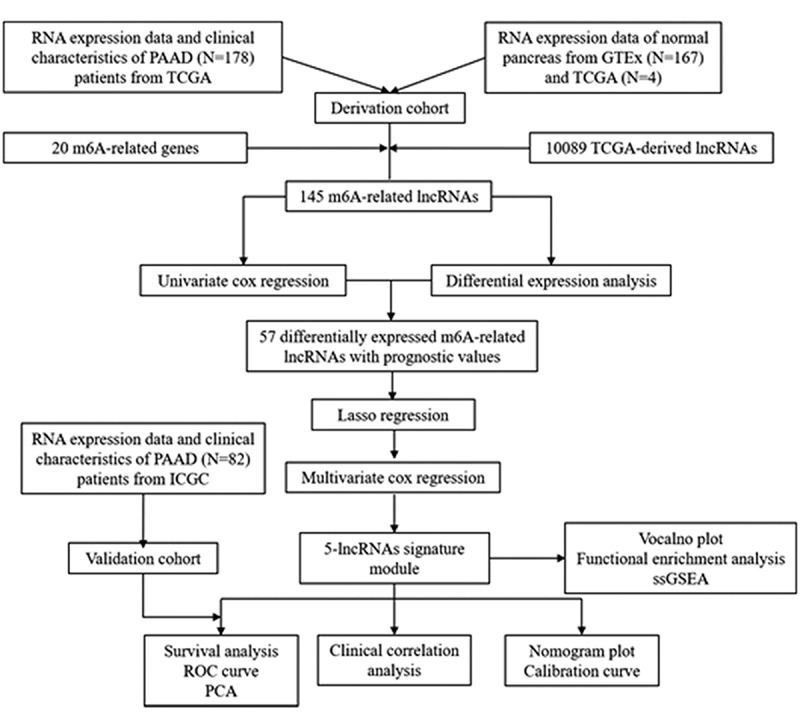

## Introduction

Pancreatic adenocarcinoma (PAAD), which originates primarily from pancreatic exocrine cells, has become the third-leading cause of cancer-related mortality in the United States [1]. Malignancy is often diagnosed at an advanced stage due to the occult location of the pancreas as a retroperitoneal organ, absence of typical clinical symptoms and lack of effective screening methods, contributing to a 5-year survival rate ranging from approximately 2% to 9% [2]. PAAD has gained considerable attention and been extensively researched in recent years, and numerous sensitive and effective methods have been proposed for its diagnosis and treatment. Nevertheless, mortality in this terrible disease continues to increase and may surpass that of colon cancer by 2030 to become the so-called ‘king’ of cancer [3]. In clinical research, it is important to focus on the underlying mechanisms of PAAD at the genetic level. In the intricate process of tumourigenesis and development, epigenetic modification, which acts as a regulator of expression of oncogenes without DNA sequence change, has received increasing attention in recent years [4].

In addition to well-established DNA and histone modifications, studies have highlighted the role of mRNA modifications in the pathogenesis of tumors. With the widespread application of gene chip and sequencing technology, it is generally accepted that such a modification process exists for mRNA splicing, nucleation, stabilization, translation and other metabolic courses, influencing gene expression. N6-methyladenosine (m6A), a methylation modification that occurs at adenine (A) in RNA, is the most common type of the 171 known RNA post-transcriptional modifications in most eukaryotic mRNAs and long non-coding RNAs (lncRNAs) and is likewise found in microRNAs, ribosomal RNAs, and transfer RNAs [5,6]. It has been reported that aberrant expression of m6A has the potential to determine the progression and prognosis of many malignancies [7–11]. Recent studies have revealed that certain m6A genes (e.g., RBM15, METTL14, FTO, and ALKBH5) are significantly associated with PAAD stage and that the expression level of ALKBH5 has a strong influence on the infiltration of CD8 + T cells in the immune microenvironment of pancreatic cancer [12,13]. An increasing number of prognostic signatures associated with m6A genes are being constructed to predict the clinical outcomes of PAAD patients.

LncRNA is a newly discovered gene regulator with a length of more than 200 transcripts that is an important part of epigenetic modification [14]. Although lncRNA do not encode proteins, they act to regulate a variety of biological processes in cells and participate in the occurrence and development of various diseases, including PAAD [15]. For example, up-regulation of LINC01232 was found to be significantly related with lower survival rates of PAAD patients, whereas its down-regulation might improve prognosis by suppressing the proliferation and migration of cancer cells [16]. In 2019, Wei et al. constructed a nine immune-related lncRNA prognostic signature to predict the prognosis of PAAD patients [17]. However, no such association of m6A-related lncRNAs and PAAD prognosis has been reported thus far. Hence, the aim of the current study was to identify and verify a novel m6A-LPS to predict PAAD patients’ survival more accurately. The goal was to provide valuable information for clinical decision-making and individual management of PAAD patients.

## Materials and methods

### Data acquisition and processing

Twenty common m6A-related genes were identified in the literature (readers: YTHDC1/YTHDC2/IGF2BP1/IGF2BP2/IGF2BP3/YTHDF1/YTHDF2/YTHDF3/HNRNPC/HNRNPA2B1/RBMX; writers: METTL3/METTL14/METTL16/WTAP/KIA1499/RBM15/ZC3H13; erasers: FTO/ALKBH5) [18–20]. Gene expression profile data and corresponding clinical information were downloaded from The Cancer Genome Atlas (TCGA) and the Genotype-Tissue Expression project (GTEx), which were considered the training dataset for constructing m6A-LPS. The training dataset consisted of 178 PAAD samples and 4 adjacent peritumoural tissues from TCGA and 167 normal pancreas tissues from GTEx. In addition, we downloaded raw data for 82 PAAD patients from the International Cancer Genome Consortium (ICGC) database; these data were used as the validation dataset for establishing the prognostic model. The ‘sva’ package in R was employed to batch normalize the gene expression profile data from different databases [21,22].

### Identification of differentially expressed m6A-related lncRNAs with prognostic value

Gene probes of the expression matrix were annotated according to the lncRNA annotation file acquired from GENCODE (https://www.gencodegenes.org/), with 18,791 protein-coding genes and 10,089 lncRNAs identified. The Pearson correlation coefficient between 20 m6A-related protein-coding genes and the 10,089 lncRNAs was computed by the built-in function ‘cor.test’ in R. Subsequently, 145 m6A-related lncRNAs with |correlation coefficient| > 0.5 and adjusted P value (adj. P) < 0.05 were selected for further analysis.

The ‘limma’ package in R was used to analyze and screen differential expression of 145 m6A-related lncRNAs between the PAAD group and the control group, with a false discovery rate (FDR) < 0.05 in the cohort from TCGA [23,24]. Univariate Cox analysis adjusted by the Benjamini & Hochberg (BH) method was implemented to explore the prognostic performances of the 145 m6A-related lncRNAs. Ultimately, 57 differentially expressed m6A-related lncRNAs with prognostic values were preserved.

### Construction and validation of a novel multi-lncRNA prognostic signature based on differentially expressed m6A-related lncRNAs with prognostic value

Least absolute shrinkage and selection operator (LASSO) regression analysis was further performed to eliminate collinearity of the 57 variables and avoid over-fitting of the constructed model [25,26]. Subsequently, multivariate Cox proportional hazards regression analysis was utilized to construct m6A-LPS and calculate risk scores using the following formula: risk score = $\overset{n}{\underset{k-1}{\sum }}\exp k*\beta k$ [4,27]. The samples were then stratified into high-risk and low-risk subgroups according to the median risk score. For both TCGA and ICGC cohorts, principal component analysis (PCA) was applied to explore the distribution of different subgroups [17]. Survival analysis with the Kaplan-Meier method was employed to evaluate the predictive ability of m6A-LPS in both cohorts. A time-dependent receiver operating characteristic (ROC) curve was plotted to verify the m6A-LPS diagnostic values of 1-year, 3-year, and 5-year survival rates using the ‘survivalROC’ package in R [28].

### Association of m6A-LPS with clinicopathological traits

To investigate the relationship between m6A-LPS and clinicopathological traits, the t-test was applied to compare discrepancy in risk scores in subgroup analyses of the following: age (<65/≥65), sex (Female/Male), race (Asian/Black or African American/White), stage (Stage I/Stage II/Stage III/Stage IV), histologic grade (G1/G2/G3/G4/GX), neoplasm location (Head of pancreas/Body of pancreas/Tail of pancreas/Other), maximum tumor dimension (<3.5/≥3.5), surgery type (Distal Pancreatectomy/Total Pancreatectomy/Whipple/Other Method), residual tumor (negative resection margins (R0)/microscopic tumor infiltration (R1)/macroscopic residual tumor (R2)), radiation therapy (Yes/No), cancer status (With tumor/Tumor free), chronic pancreatitis history (Yes/No), diabetes history (Yes/No), drinking frequency (None/Daily Drinker/Occasional Drinker/Social Drinker/Weekly Drinker), smoking history (I/II/III/IV/V), and family history of cancer (Yes/No).

### Co-expression status of m6A genes and related lncRNAs and clinical significance

After verifying the effectiveness of m6A-LPS for predicting the prognosis of PAAD patients, a Sankey diagram and correlation circle graph were applied to demonstrate the co-expression status of m6A genes and their related lncRNAs using the gene expression profiles of the cohort from TCGA [29]. Subsequently, the chi-square test was employed to explore correlation between these m6A genes as well as their related lncRNAs and the stage and grade of PAAD patients.

### Independent prognostic performance of m6A-LPS in the cohort from TCGA

To analyze whether m6A-LPS can serve as an independent prognostic indicator, univariate and multivariate Cox proportional hazards regression analyses were performed using risk scores and clinicopathological parameters. Next, factors significantly associated with the prognosis of PAAD patients in both univariate and multivariate analyses (p < 0.05) were selected to plot a nomogram for the cohort from TCGA using the ‘rms’ R package; calibration curves for 0.5-year, 1-year, and 2-year survival rates in the cohort were also plotted to examine the degree of fitting between nomogram-estimated and actual survival probabilities [30].

### Exploration of cellular processes, signaling pathways, and immune status affected by m6A-LPS

To explore the potential mechanisms of different prognoses between high-risk and low-risk subgroups, the ‘limma’ R package was utilized to determine differentially expressed genes (DEGs, |log2FC| > 1 and FDR < 0.05) between the subgroups. Functional enrichment analysis of DEGs was further carried out to assess discrepancy in biological processes (BP), cellular components (CC), molecular functions (MF), and signaling pathways between the high-risk and low-risk subgroups [31]. In addition, we performed single-sample gene set enrichment analysis (ssGSEA) based on the ‘gsva’ R package to evaluate disparity in immune cells and immune-related functions between these subgroups [32]. All statistical analyses in our study were conducted based on the R language.

## Results

In the current study, we aimed to construct and validate a novel m6A-LPS for better prediction of the prognosis of patients with PAAD. We identified 57 differentially expressed m6A-related lncRNAs with prognostic values, and these lncRNAs were used to construct and validate a novel prognostic model for patients with PAAD based on LASSO-Cox regression analysis. In addition, we performed functional and single-sample gene set enrichment analyses to explore the different cellular processes, signaling pathways, and immune status between high- and low-risk groups.

### Identification of differentially expressed m6A-related lncRNAs with prognostic values

**Figure 1.**
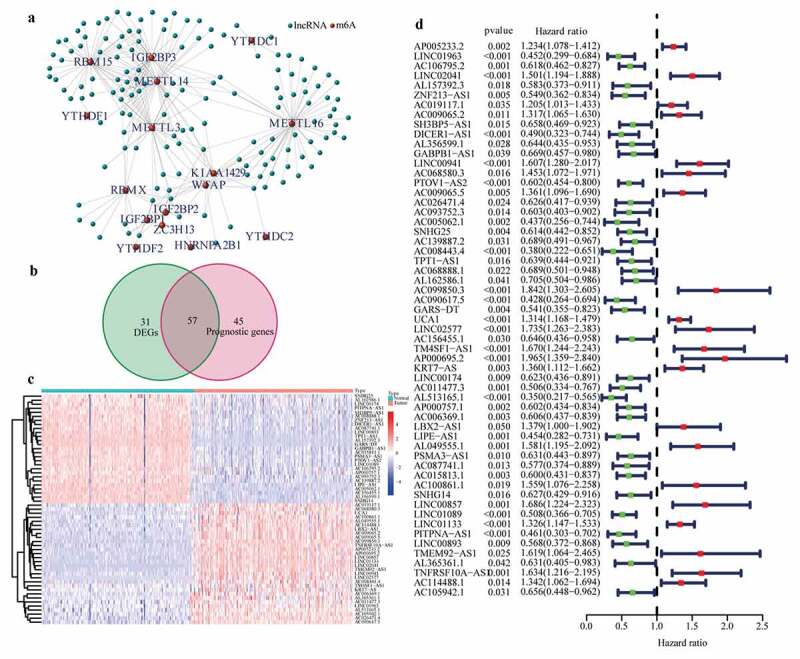
Identification of the differentially expressed m6A-related lncRNAs with prognostic value in the cohort from TCGA. (a) Identification of m6A-related lncRNAs (b) Venn diagram to identify differentially expressed m6A-related lncRNAs between PAAD and normal pancreas tissues associated with prognosis. (c) Heatmap to explore mRNA levels of 57 differentially expressed m6A-related lncRNAs with prognostic values. (d) Univariate Cox regression analysis of 57 differentially expressed m6A-related lncRNAs with prognostic values

Expression profiles of 20 common m6A genes and 10,089 lncRNAs were obtained by analyzing 178 PAAD samples from TCGA and 171 normal pancreas samples (comprising 4 and 167 normal samples from TCGA and GTEx, respectively). A total of 145 m6A-related lncRNAs emerged through the Pearson test (Figure 1(a)) and were selected to conduct differential expression analysis and univariate Cox regression analysis. 57 differentially expressed m6A-related lncRNAs with prognostic values were preserved (Figure 1(b)). The results of differential expression analysis and univariate Cox regression analysis of these 57 lncRNAs are shown in (Figure 1(c,d)).

### Construction of the m6A-LPS based on the cohort from TCGA

**Figure 2.**
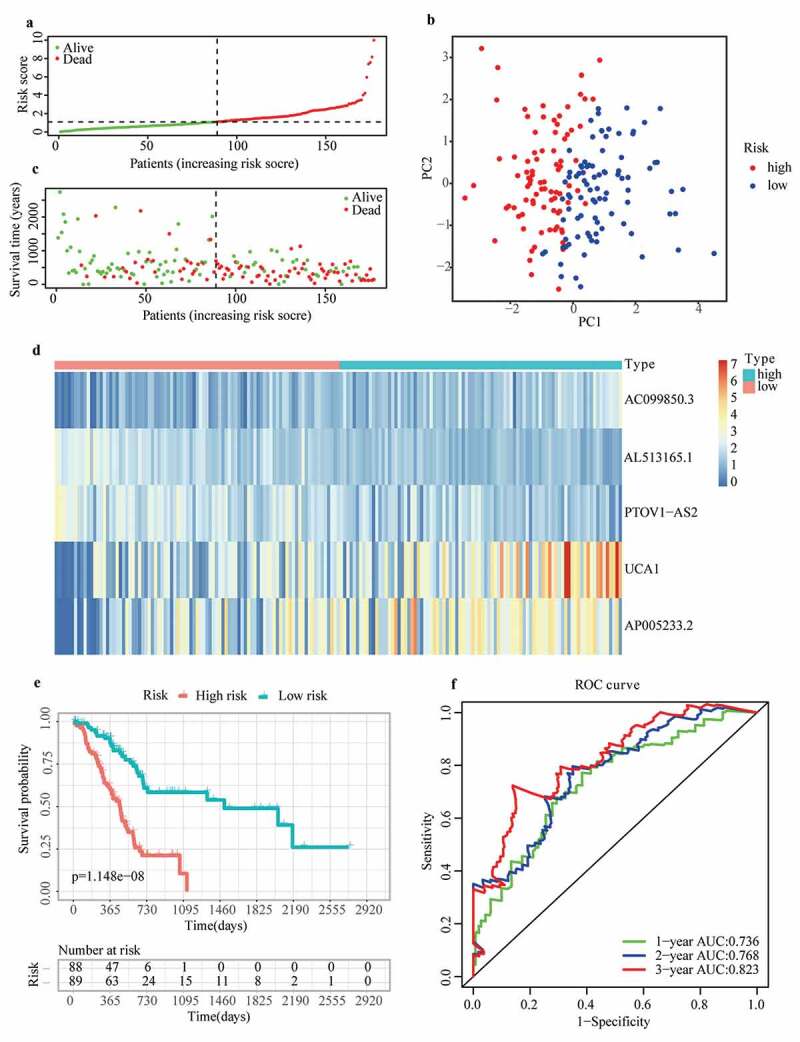
Prognostic performance of the m6A-LPS in the cohort from TCGA. (a) The distribution and median value of the risk scores in the cohort from TCGA. (b) PCA plot of the cohort from TCGA. (c) Distributions of OS status, OS and risk score in the cohort from TCGA. (d) Differential expression of five lncRNAs used for constructing m6A-LPS between high- and low-risk subgroups. (e) Kaplan-Meier curves for the OS of patients in the high- and low-risk groups in the cohort from TCGA. (f) AUC of the time-dependent ROC curve validated the prognostic value of the risk score in the cohort from TCGA

To eliminate collinearity of the variables and avoid over-fitting of the prognostic model, LASSO regression analysis was carried out for 57 differentially expressed m6A-related lncRNAs with prognostic values, and 10 lncRNAs were obtained for further multivariate Cox regression analysis (Appendix A1 and A2). Ultimately, m6A-LPS was established to predict the prognosis of PAAD patients based on the expression values of five lncRNAs (AC099850.3, UCA1, AP005233.2, AL513165.1, and PTOV1-AS2). As shown in (Figure 1(d)), higher expression levels of AC099850.3 (HR = 1.842, 95%CI = 1.303 − 2.605), UCA1 (HR = 1.314, 95%CI = 1.168 − 1.479) and AP005233.2 (HR = 1.234, 95%CI = 1.078 − 1.412) were associated with poorer prognoses in PAAD patients. In contrast, higher expression levels of AL513165.1 (HR = 0.350, 95%CI = 0.217 − 0.565) and PTOV1-AS2 (HR = 0.602, 95%CI = 0.454 − 0.800) were significantly associated with better prognosis in PAAD. Then, based on the Cox coefficient of five lncRNAs for modeling, a prognostic risk score was computed for each patient in the cohort from TCGA, as follows: (0.3068541 × expression level of AC099850.3) + (0.155491381 × expression level of UCA1) + (0.243316592 × expression level of AP005233.2) − (0.71041932 × expression level of AL513165.1) − (0.647340922 × expression level of PTOV1-AS2). Subsequently, all patients were stratified into high- and low-risk subgroups based on their median risk score (Figure 2(a)). According to PCA, the patients of different subgroups could be distinguished clearly on PC1 and PC2 (Figure 2(b)). The distributions of the risk score and survival status are presented in (Figure 2(c)). Mortality increased with a higher risk score. In the high-risk subgroup, AC099850.3, UCA1 and AP005233.2 were expressed at higher levels, whereas AL513165.1 and PTOV1-AS2 were expressed at lower levels (Figure 2(d)). According to the survival curves depicted in (Figure 2(e)), the high-risk subgroup showed a poorer overall survival rate than the low-risk subgroup. In (Figure 2(f)), ROC curves for TCGA cohort data revealed AUC values of 0.736, 0.768 and 0.823 for 1-, 2- and 3-year survival, respectively, which indirectly reflects that m6A-LPS can strongly predict 1-, 2-, and 3-year survival rates for PAAD patients.

### Validation of m6A-LPS based on the cohort from ICGC

**Figure 3.**
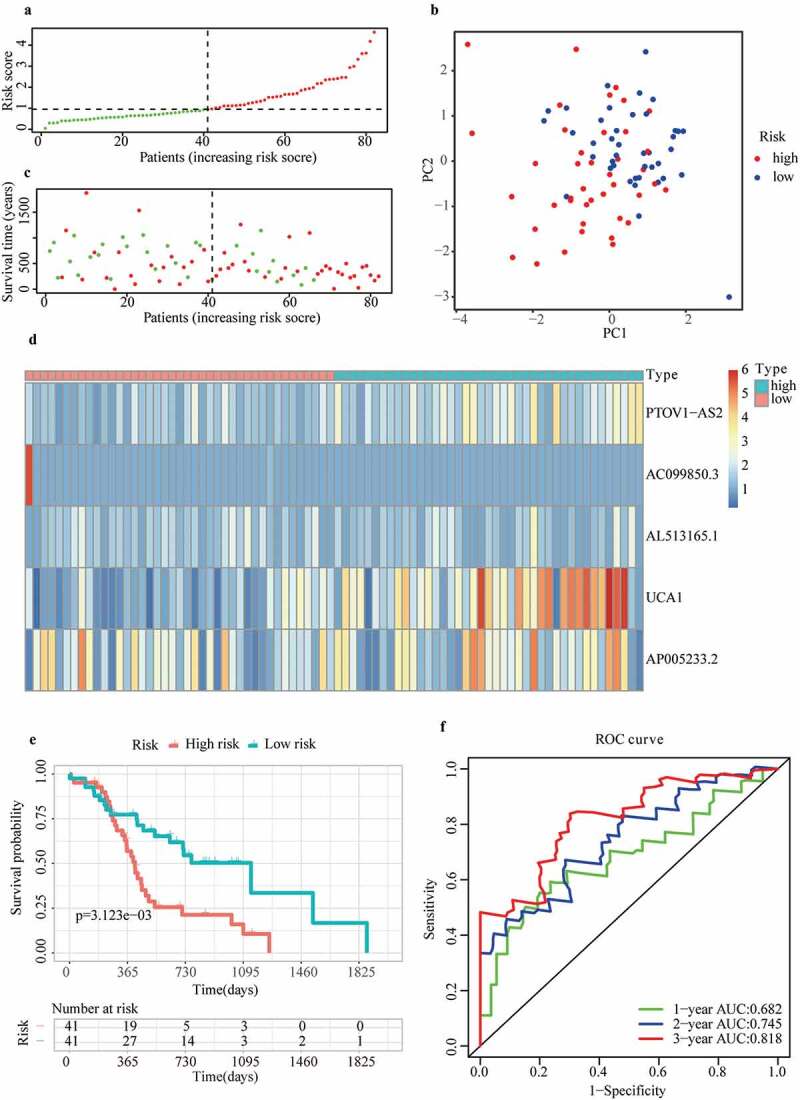
Prognostic performance of m6A-LPS in the cohort from ICGC. (a) The distribution and median value of the risk scores in the cohort from ICGC. (b) PCA plot of the cohort from ICGC. (c) Distributions of OS status, OS and risk score in the cohort from ICGC. (d) Differential expression of five lncRNAs used for constructing m6A-LPS between high- and low-risk subgroups. (e) Kaplan-Meier curves for the OS of patients in the high- and low-risk groups in the cohort from ICGC. (f) AUC of the time-dependent ROC curve validated the prognostic value of the risk score in the cohort from ICGC

To verify the predictive reliability of m6A-LPS, 82 samples in the cohort from ICGC were also stratified into high- and low-risk subgroups according to the median risk score, and the calculation formula of the risk score was the same as that for the cohort from TCGA (Figure 3(a)). The PCA results in (Figure 3(b)) show that the distribution of the two subgroups in the cohort from ICGC was similar to that in the cohort from TCGA. In addition, patients in the high-risk subgroup had a worse survival status (Figure 3(c)), and discrepancy in the expression level of 5 lncRNAs for modeling between high- and low-risk subgroups indicated conclusions similar to that for the cohort from TCGA (Figure 3(d)). Likewise, according to the survival curves depicted in (Figure 3(e)), the high-risk subgroup also showed a poorer overall survival probability than the low-risk subgroup. ICGC cohort analysis also showed great predictive accuracy for m6A-LPS. As illustrated in (Figure 3(f)), the AUC of m6A-LPS was 0.682 at 1 year, 0.745 at 2 years, and 0.818 at 3 years for the ICGC cohort.

### Association of the risk score acquired from m6A-LPS and clinicopathological traits

**Figure 4.**
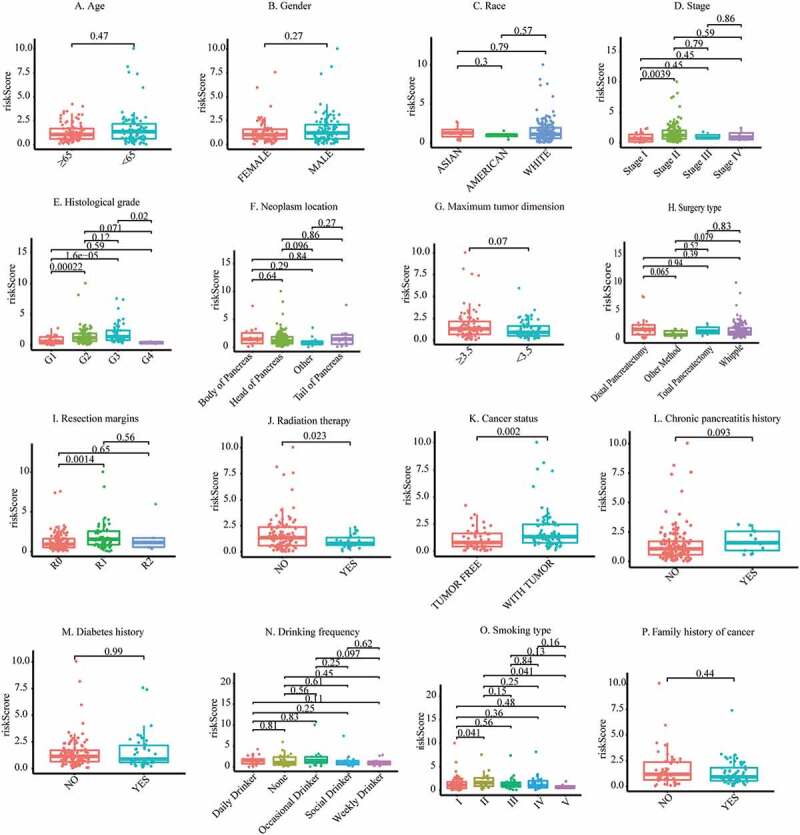
Discrepancy in risk scores between different subgroups: (a) Age, (b) Sex, (c) Race, (d) Stage, (e) Histological grade, (f) Neoplasm location, (g) Maximum tumor dimension, (h) Surgery type, (i) Resection margins, (j) Radiation therapy, (k) Cancer status, (l) Chronic pancreatitis history, (m) Diabetes history, (n) Drinking frequency, (o) Smoking type, (p) Family history of cancer

To better understand the clinical significance of m6A-LPS in PAAD, we compared discrepancies in risk scores between different subgroups of PAAD (Figure 4). The subgroup analysis stratified by stage demonstrated a significantly elevated risk score in stage II PAAD patients compared to stage I PAAD patients (p = 0.0039). Stratification by histological grade revealed a significantly increased risk score in G2 (p = 0.00022) and G3 (p = 1.6e−05) PAAD patients compared to G1 PAAD patients. Moreover, a significantly reduced risk score was detected in G4 PAAD patients compared to G3 PAAD patients (p = 0.02). Of note, the number of samples in the G4 subgroup were so small that the results regarding G4 should be considered with caution. In subgroup analysis stratified by the maximum tumor dimension, a trend toward a higher risk score in the ≥3.5 cm subgroup did not achieve statistical significance (p = 0.07). The risk score for PAAD patients treated with distal pancreatectomy (p = 0.065) and the Whipple (p = 0.079) procedure exhibited an increasing tendency compared to that in PAAD patients treated with other surgery types. PAAD patients with R0 resection had a significantly lower risk score than those with R1 resection (p = 0.0014), and those treated with radiation therapy had a significantly lower risk score than those not given radiation therapy (p = 0.023). PAAD patients who still had tumors after surgery had a higher risk score than PAAD patients who were tumor free after surgery (p = 0.002). Furthermore, PAAD patients with a history of chronic pancreatitis showed a definitely increased risk score compared to those without a chronic pancreatitis history (p = 0.093). PAAD with smoking history of type II had a significantly higher risk score than those with type I (p = 0.041). However, there was no significant association between age, sex, race, neoplasm location, diabetes history, alcohol consumption frequency, family history of cancer and risk score.

### Co-expression status of m6A genes and related lncRNAs and clinical significance

**Figure 5.**
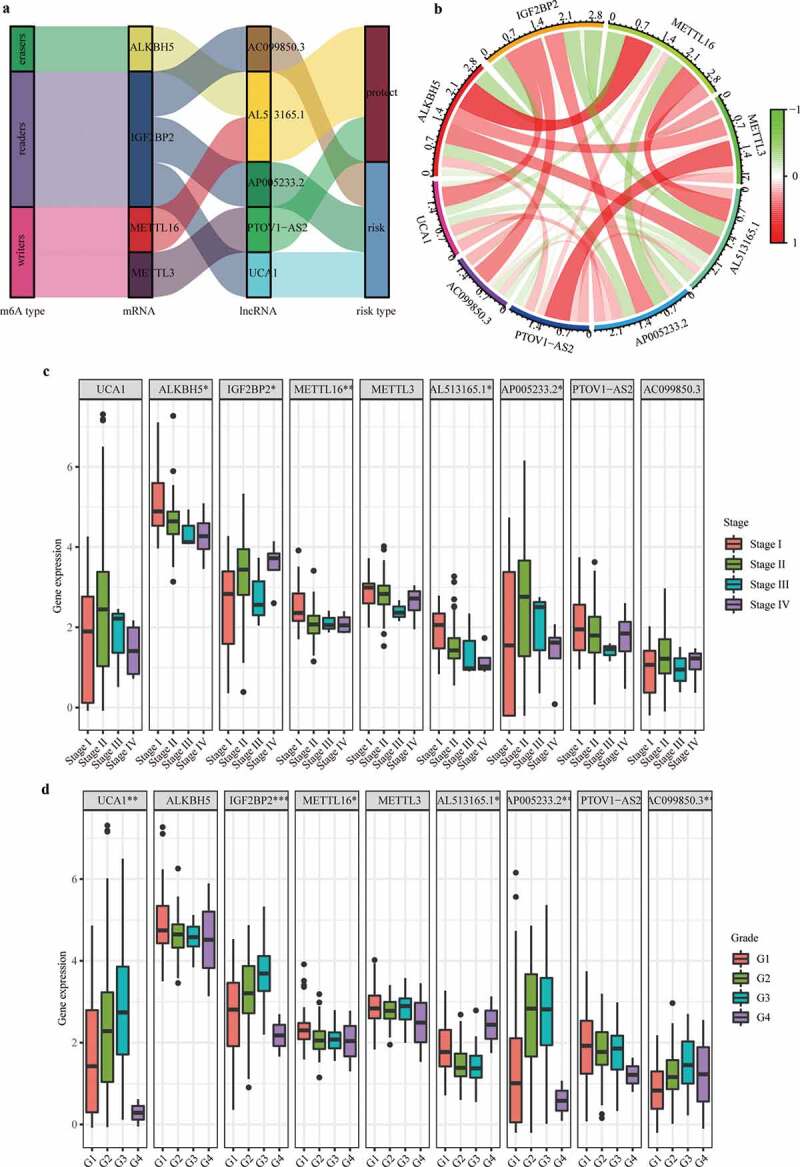
Co-expression status of m6A genes and their related lncRNAs and clinical significance. (a) Sankey plot to identify a one-to-one match between m6A genes and their related lncRNAs. (b) Circle plot for the correlation between m6A genes and their related lncRNAs. (c) Differential expression of m6A genes and their-related lncRNAs between stage I/II/III/IV patients. (d) Differential expression of m6A genes and their related lncRNAs between grade I/II/III/IV patients

Under the threshold of |Pearson correlation coefficient| > 0.5 and adj. P < 0.05, 4 m6A genes (i.e., writers: METTL16 and METTL3; readers: IGF2BP2; erasers: ALKBH5) were detected to be co-expressed with 5 lncRNAs used for constructing m6A-LPS. As depicted in (Figure 5(a)), a Sankey diagram demonstrated a one-to-one match between these 4 m6A genes and 5 m6A-related lncRNAs and the risk type of each lncRNA (protective lncRNAs: AL513165.1 and PTOV1-AS2; risk lncRNAs: AC099850.3, UCA1, and AP005233.2). Moreover, a correlation circle plot demonstrated the co-expression status between these 4 m6A genes and 5 m6A-related lncRNAs (Figure 5(b)). The boxplots in (Figure 5(c,d)) also show the differential expression of these 4 m6A genes and 5 m6A-related lncRNAs among stage I/II/III/IV patients and grade I/II/III/IV patients, respectively. The results suggest that expression of ALKBH5, IGF2BP2, METTL16, AL513165.1, and AP005233.2 is significantly associated with tumor stage and that expression of UCA1, IGF2BP2, METTL16, AL513165.1, AP005233.2, and AC099850.3 is significantly associated with tumor grade.

### Independent prognostic performance of m6A-LPS in the cohort from TCGA

**Figure 6.**
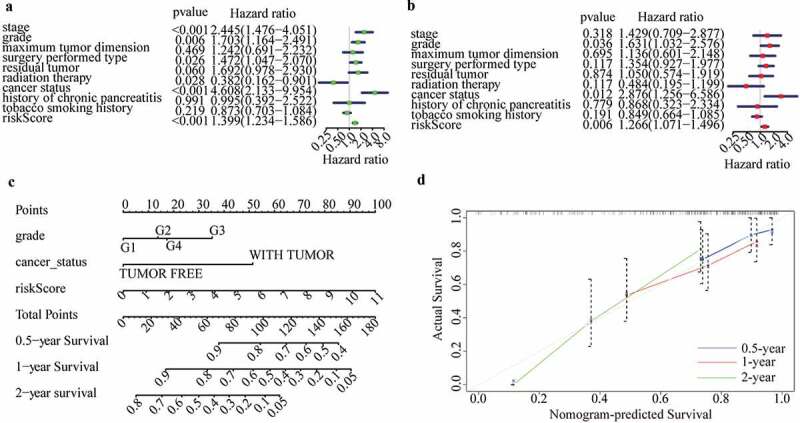
Independent prognostic analysis of the risk score acquired from m6A-LPS in the cohort from TCGA. (a) Univariate and (b) multivariate Cox regression analyses of the association between risk score, clinicopathological parameters and overall survival of patients in the cohort from TCGA. (c) Nomogram composed of grade, cancer status and risk score for the prediction of 0.5-, 1-, and 2-year OS probability. (d) Calibration plot for the evaluation of the nomogram in predicting 0.5-year, 1-year, and 2-year OS probability

The clinicopathological parameters associated with the risk score acquired from the m6A-LPS were selected for univariate and multivariate Cox regression analyses. The results showed that risk score, histological grade, and cancer status were all independent prognostic predictors (univariate HR = 1.397, 95%CI = 1.236 − 1.578, p < 0.001; multivariate HR = 1.276, 95%CI = 1.075 − 1.516, P < 0.05; (Figure 6(a,b)). Subsequently, to quantitatively predict the survival probability of each PAAD patient, a nomogram for 0.5-year, 1-year, and 2-year survival rates was plotted based on the risk score, histological grade, and cancer status of each PAAD patient (Figure 6(c)). Moreover, calibration curves for 0.5-year, 1-year, and 2-year survival rates were plotted to verify the accuracy of the nomogram, with the results indicating general agreement between the nomogram-predicted and actual survival probabilities (Figure 6(d)).

### Exploration of cellular processes, signaling pathways, and immune status affected by m6A-LPS

**Figure 7.**
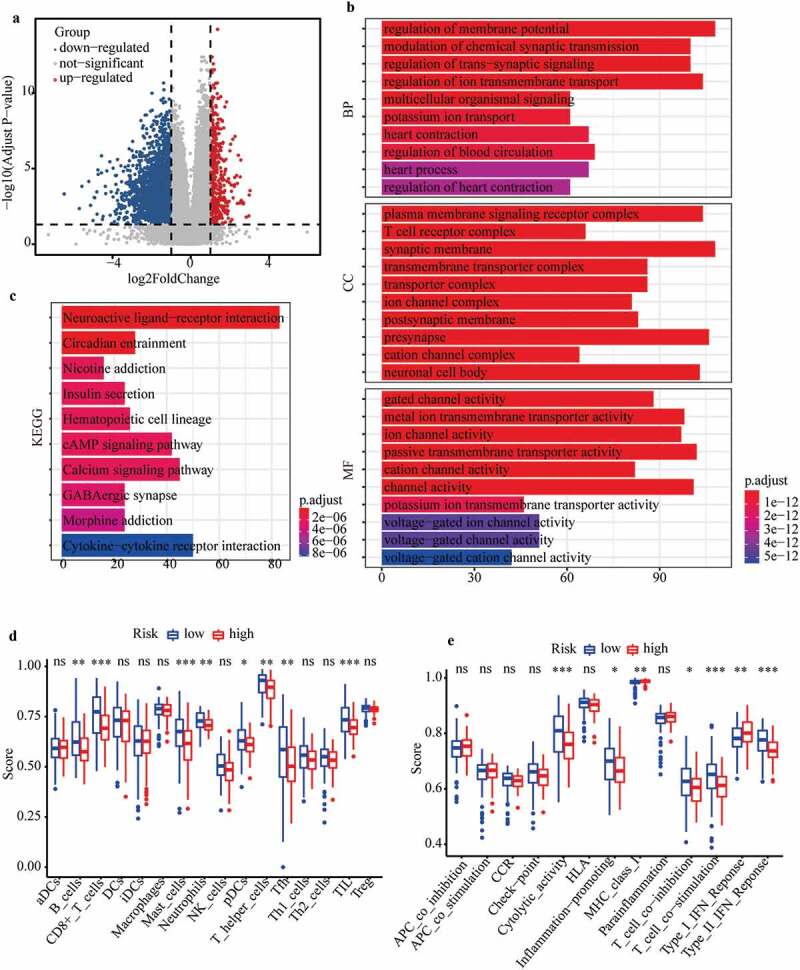
Investigation of cellular processes, signaling pathways, and immune status affected by m6A-LPS. (a) Volcano plot of DEGs between high- and low-risk groups. (b) GO enrichment and (c) KEGG pathway analysis of DEGs between high- and low-risk groups. Comparison of ssGSEA scores between different risk subgroups. The scores for (d) 16 immune cells and (e) 13 immune-related functions are displayed in boxplots

A total of 3194 DEGs (comprising 442 up-regulated and 2752 down-regulated) between the high- and low-risk subgroups were evaluated to explore the cellular processes and signaling pathways affected by m6A-LPS (Figure 7(a)). Gene Ontology (GO) functional enrichment analysis was applied to annotate DEG functions, and several cancer-related BPs were identified, including modulation of chemical synaptic transmission and regulation of ion transmembrane transport. In addition, CC results indicated that the DEGs are mainly involved in the T cell receptor complex, transmembrane transporter complex and ion channel complex. MF results showed that the DEGs play a pivotal role in a variety of channel activities, such as ion channel activity and passive transmembrane transporter activity (Figure 7(b)). Kyoto Encyclopedia of Genes and Genomes (KEGG) analysis identified several pathways in which DEGs were enriched, including insulin secretion, cAMP signaling pathway, calcium signaling pathway and cytokine−cytokine receptor interaction (Figure 7(c)).

Additionally, ssGSEA was conducted to explore discrepancies in the immune status and function between the high- and low-risk subgroups. Interestingly, the majority of immune cell scores in the high-risk subgroup showed a decreasing tendency (Figure 7(d)). Of note, T cell-mediated specific anti-tumor immunity (comprising cytotoxic T lymphocytes (CTLs) and T helper (Th) cells) differed significantly between the high- and low-risk subgroups (Figure 7(d)). Additionally, scores of CD8 + T cells and Th cells were significantly lower in the high-risk subgroup than in the low-risk subgroups, indicating that the ability to kill tumor cells was weaker in the former (Figure 7(d)). The scores for B cells and follicular helper T (Tfh) cell-mediated humoural immunity were significantly lower in the high-risk subgroup than in the low-risk subgroup (Figure 7(d)). Similarly, the tumor infiltrating lymphocyte (TIL) score (including T lymphocytes and B lymphocytes) was significantly lower in the high-risk subgroup (Figure 7(d)). Moreover, immune-related functions differed significantly between the high- and low-risk subgroups (Figure 7(e)). The cytolytic activity score (CYT), as a practical tool to evaluate antitumour immunity, was significantly lower in the high-risk subgroup, which indirectly indicated that CTL function was weaker in this subgroup (Figure 7(e)). Scores for the inflammation-promoting response and type II interferon (IFN) response were significantly lower in the high-risk subgroup, though the opposite was found for the type I IFN response score (Figure 7(e)).

## Discussion

PAAD is a common digestive system malignant tumor with high mortality. It is generally accepted that various intertwined factors, including genetic susceptibility and environmental stimuli (such as cigarettes, diabetes mellitus, and adiposity), are potential factors of morbidity and mortality in patients with PAAD [33,34]. From the perspective of genes, m6A, as one of the prevailing epigenetic modifications occurring post-transcriptionally, plays crucial roles in various diseases, including cancers. With the completion of the Human Genome Project, it has been found that only 2% of the total number of genes encode and express proteins, with more than 90% being transcribed into non-coding RNAs, which have important roles in the complex functional regulation of life [35]. Among them, lncRNAs are transcripts longer than 200 nucleotides without protein-coding ability [36]. Microarray and high-throughput sequencing technologies have revealed a large number of lncRNAs in the human body, and further studies are gradually proving that lncRNAs play important regulatory roles in various physiological or pathological processes. For instance, abnormal expression of lncRNAs is closely related to the occurrence and development of Alzheimer’s disease, heart disease, cancers and many other diseases [37–39]. Nevertheless, the potential role and prognostic performance of m6A-related lncRNAs in PAAD remains unclear.

In our research, we intensively explored differential expression of m6A-related lncRNAs between PAAD samples and normal pancreas tissues and revealed the prognostic performances of m6A-related lncRNAs in PAAD. More importantly, we identified and verified a novel prognostic signature based on expression of differentially expressed m6A-lncRNAs with prognostic values. PAAD patients were stratified into high- and low-risk subgroups with different clinical outcomes. ROC curves for 1-year, 2-year, and 3-year survival rates validated the predictive accuracy of m6A-LPS. Furthermore, t-test results revealed that risk scores (computed by: 0.307*AC099850.3 +0.155*UCA1 + 0.243*AP005233.2 − 0.71*AL513165.1 − 0.647*PTOV1-AS2) acquired from m6A-LPS were associated with tumor stage, histological grade, maximum tumor dimension, surgery type, resection margin, radiation therapy, cancer status, chronic pancreatitis history, and smoking habitats. Univariate and multivariate Cox regression analyses proved that our risk score could serve as an independent prognostic index, indirectly indicating the independent prognostic performance of m6A-LPS. Then, a precise nomogram comprising risk score, histological grade, and cancer status was identified and validated by calibration curves. Functional enrichment analysis and ssGSEA were performed to explore discrepancy in cellular processes, signaling pathways, and immune status between high- and low-risk subgroups.

It is well established that the main immune anti-tumor mechanisms are as follows. First, CD8 + T lymphocytes have the potential to activate, proliferate and differentiate into effector CTLs after stimulation by tumor-associated antigens. Subsequently, chemokines promote CTL migration from peripheral immune organs into the tumor site and killing of tumor cells through the perforin-granzyme, Fas-FasL, and TNF-TNFR pathways. CYT is a useful measurement tool that can indirectly reflect the destruction of tumor cells by CTLs. Second, Th cells not only play auxiliary role in the activation of CTLs but also produce cytokines that indirectly participate in anti-tumor immune effects, such as secreting IFN to activate macrophages and enhance their phagocytosis and killing effect on tumor cells and secreting TNF to induce tumor cell apoptosis and tumor vascular necrosis. Third, innate immune cells, including macrophages, NK cells and NKT cells, also play an irreplaceable role in anti-tumor immunity. Fourth, tumor antigens stimulate B lymphocytes to differentiate into plasma cells and secrete antibodies with anti-tumor effects. Interestingly, the high- and low-risk subgroups produced by m6A-LPS showed significantly different immune cell infiltrations and immune function scores. Specifically, the scores for CD8 + T cells, Th cells, B cells, and CYT were significantly lower in the high-risk subgroup, indicating that anti-tumor capacity in this subgroup was weaker than that in the low-risk subgroup. However, no significant difference in innate immune cell scores between the high- and low-risk subgroups was observed. Overall, the impaired anti-tumor ability in the high-risk subgroup greatly indicated the poorer prognosis of this subgroup.

Our m6A-LPS consists of five prognostic lncRNAs associated with m6A genes (i.e., AC099850.3, UCA1, AP005233.2, AL513165.1 and PTOV1-AS2). AC099850.3 is related to m6A genes and is also closely related to autophagy genes. AC099850.3 is significantly related to overall survival in squamous cell carcinoma of the tongue; it was also selected as a member of a predictive signature in hepatocellular carcinoma [40–42]. Abnormal expression of UCA1 is reported to be related to the occurrence and development of various cancers [43,44]. Specifically, UCA1 is considered a sensitive and specific biomarker for bladder cancer and a promising therapeutic target for colorectal cancer [45,46]. Abnormal expression of UCA1 promotes the invasion and metastasis of ovarian cancer cells by up-regulating matrix metalloproteinase (MMP) 2 and MMP9 [47–50]. In addition, high expression of AP005233.2 was significantly associated with poorer prognoses of non-small-cell lung cancer (NSCLC), and it might participate in the tumourigenesis of NSCLC by binding with cyclin D1 [51]. The remaining two key lncRNAs have rarely been reported.

Despite the important findings of our research, the present study has some limitations that should be considered. First, due to the limitations of the databases TCGA and ICGC, relatively few pancreatic adenocarcinoma samples were included in our study, and more high-quality cohort data with large samples are needed for verification of the prognostic value of our m6A-LPS in the future. Second, we explored the potential mechanisms of different prognoses between high- and low-risk subgroups by pure bioinformatics analysis; however, the results need to be verified by more fundamental experiments.

### Conclusion

In conclusion, we successfully developed and validated a novel m6A-LPS for PAAD patients, which might provide a new perspective for clinical decision-making and precision medicine. More importantly, our study sheds new light on the potential association of m6A-related lncRNAs and tumor immunity.

## Data Availability

The datasets analysed during the current study are publicly available on the TCGA (https://cancergenome.nih.gov/), GTEx (https://www.gtexportal.org/home/) and ICGC (https://dcc.icgc.org/) databases. The data that support the findings of this study are also available from the first author (Qihang Yuan) upon reasonable request.

## Article highlights

1. We firstly develop a m6A-related multi-lncRNA risk signature for predicting survival of PAAD.
2. The novel risk signature could help clinicians to stratify PAAD patients with poor prognosis.
3. The findings provide new insight into clinical decision-making and precision medicine of PAAD.

## Appendix A.

LASSO regression analysis. (A1) LASSO regression analysis to eliminate collinearity. (B) Partial likelihood deviance for different numbers of variables.
